# Effect of vehicle external acceleration signal light on pedestrian-vehicle interaction

**DOI:** 10.1038/s41598-023-42932-2

**Published:** 2023-09-28

**Authors:** Feng Li, Wenjun Pan, Jiali Xiang

**Affiliations:** School of Art and Design, Zhejiang Sci-Tech University, Hangzhou, 310018 China

**Keywords:** Environmental social sciences, Engineering

## Abstract

The number of casualties resulting from collisions between pedestrians and motor vehicles continues to rise. A significant factor is the misunderstanding of vehicle behavior intentions by pedestrians. This is especially true with the continuous development of vehicle automation technology, which has reduced direct interaction between drivers and the outside world. Therefore, accurate communication of vehicle behavior intentions is becoming increasingly important. The purpose of this study is to investigate the impact of external vehicle acceleration signal light on the interaction experience between pedestrians and vehicles. The differences between the use and nonuse of acceleration signal light are compared through controlled test track experiments in real scenarios and in videos.The results show that acceleration signal light help pedestrians understand vehicle behavior intentions more quickly and make safer crossing decisions as well as improving their perception of safety when crossing the street and their trust in vehicle behavior.

Pedestrians are the most vulnerable of all road users and are the group with the largest safety risks^1^. According to the 2018 Global Road Safety Status Report released by the World Health Organization (WHO), approximately 1.35 million people die from road traffic accidents worldwide every year, among which 23% are pedestrians, and more than 80% of pedestrian accidents are caused by motor vehicle collisions^2^. Especially in road sections with no traffic light, the ownership of the road between pedestrians and vehicles is not clear; this leads to mutual rush and increasingly serious conflicts between pedestrians and vehicles^3^. The main reason for this conflict lies in the lack of communication or misunderstanding between vehicles and pedestrians^4^. Currently, many issues have been unaddressed by previous studies.

For the traditional human-driven car, communication between the vehicle and a pedestrian essentially occurs between the pedestrian and the driver of the car. The car spatially hampers communication between drivers and pedestrians, which can be better addressed by considering the car itself as a medium to connect the two parties^5^. At the same time, the interaction between pedestrians and vehicles has become more important with the rapid development and gradual widespread adoption of autonomous driving technologies. To address this issue, most research has focused on using external human‒machine interface to the vehicle to communicate with pedestrians^6^.

This study aims to create a viable solution for vehicle–pedestrian interaction by designing vehicle acceleration signal light as an external human‒machine interface on the vehicle that can provide efficient communication and solve the challenge of vehicle–pedestrian interaction. First, acceleration signal light transmit information in color in an intuitive way and are easily transferable to nonautonomous vehicles^7^. Second, acceleration signal light can display signals (red, green and white light) for different acceleration states of the vehicle (acceleration, deceleration and constant speed), which can effectively communicate vehicle information to pedestrians. This study asks the following questions about this solution: Can unclear pedestrian-vehicle interactions be improved when vehicles are equipped with acceleration signal light? Do acceleration signal light have an impact on the emotional experience of pedestrians? The aim of this study is to investigate the effect of acceleration signal light on improving the experience of pedestrians when interacting with vehicles and to enhance pedestrians’ sense of safety and trust when crossing the street to enhance road safety.

## Current status of vehicle–pedestrian interactions

The ability of pedestrians and vehicles to avoid each other reasonably and safely depends on the effectiveness and flexibility of the interaction between pedestrians and vehicles^8^. When pedestrians cross the road, the paths of movement of pedestrians and vehicles come into conflict. In the absence of traffic light, the allocation of the road right of way becomes contentious. From the pedestrian’s point of view, he or she will make a reasonable assessment based on vehicle distance, speed, size and even road conditions^9^. Having been granted the right of way by a vehicle, pedestrians will simply cross the road quickly, whereas without confirmation of the right of way attribution, pedestrians may attempt to cross the road and observe the vehicle’s reaction in real time. Thus, in the absence of a clear command signal roadway, the right of way is ambiguous. Both pedestrians and vehicles need to change their traffic decisions in real time depending on each other, and each change in traffic decisions increases road risk and may even cause traffic accidents^10^.

A large number of studies have attempted to address this problems by proposing methods to improve traffic regulations^11^, regulate driver behavior^12^, ensure vehicle driving safety and enhance infrastructure traffic facilities^13^. These measures are mainly focused on the driver’s perspective; however, research to understand vehicle and driver behavior from the pedestrian’s perspective is equally important^14^. Drivers can infer whether a pedestrian is aware of an approaching vehicle from changes in the pedestrian’s body posture or direction of gaze. In contrast, pedestrians have no reliable cues to predict the behavior of vehicles^15^.

**Figure 1:**
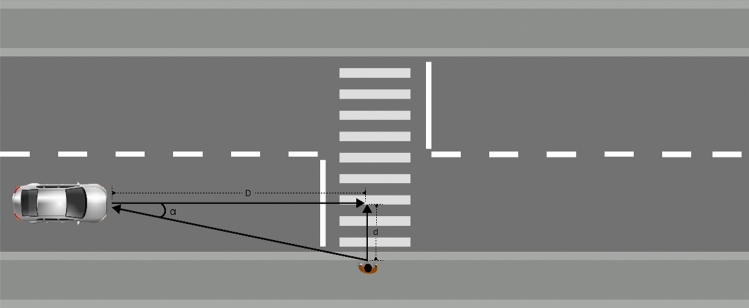
Vehicle and pedestrian conflict scenario.

To ensure their safety, pedestrians often seek to make eye contact with drivers as they prepare to cross the road. This aims to alert the driver of their presence and to understand the vehicle’s intention to move. However, in today’s traffic environment, it is almost impossible for the act of eye contact to achieve efficient interaction^16^. Studies have shown that over 90% of pedestrians cannot see the driver from 30 m away and cannot make eye contact with the driver from 15 m away^17^. In addition, pedestrians can judge vehicle behavior by sensing vehicle speed^18^. As shown in the scenario in Fig. 1, the pedestrian’s line of sight forms an angle α with the vehicle’s forward direction. From the principle of visual perception^19^, it is known that as the pedestrian walks toward the zebra crossing, the smaller the angle α becomes, and the more difficult it is for the pedestrian to perceive the vehicle’s speed. To cope with the lack of clarity in the right-of-way between pedestrians and vehicles and the difficulty for pedestrians to judge the behavior of vehicles, research on external interfaces for vehicles has gained attention. Effective external interfaces have been shown to be beneficial for pedestrian-vehicle communication^20^.

## eHMI- vehicle front signal light

eHMI (external human‒machine interface) can communicate information such as vehicle status or intentions to pedestrians or other road users for interaction between the vehicle and the external environment (including pedestrians or other vehicles)^21^ to enhance traffic safety. Related research has confirmed that eHMI complements vehicles’ kinematic cues and provides feedback on drivers’ driving intentions^22^. The main forms of eHMI include signal light, text, icons, and projections. However, there is still no consensus among relevant studies regarding which form is easier to use for pedestrian-vehicle communication^23^. Signal light and text messages are the most common forms. Text messages are relatively more complex, require larger displays on the vehicle and are difficult to apply in nonautonomous vehicles. Complex signals with too much text may act as additional stimuli, placing an additional cognitive load on pedestrians and potentially resulting in poor decision-making^24^. Therefore, the use of signal light seems to be a more reasonable solution.

The idea of using vehicle signal light to send vehicle information to other road participants emerged at an early stage, with a 1920 patent in which researchers used vehicle front signals to give feedback on the braking status of a vehicle^25,26^. At the time, rear brake light could be considered a new technology. In a subsequent study, the authors found that the majority of subjects agreed that vehicle front signal light were necessary to enhance communication between drivers and between drivers and pedestrians. Especially at night when the light is not good, the message of front signal light is more obvious^27^. One participant even reported in an interview that the brake pedal was sometimes deliberately depressed to trigger the front signal light to communicate to other road users the intention to travel. In a recent study, researchers conducted a laboratory video experiment to assess the effectiveness of front signal light for pedestrians to recognize vehicle deceleration. When the driver applied the brakes, the vehicle’s front signal light were shown in green to alert pedestrians. The results showed that the front signal light helped pedestrians recognize vehicle deceleration effectively and more quickly^7^. Vehicle front signal light have been shown to be effective in helping other road users recognize vehicle status and can help pedestrians determine whether an approaching vehicle will yield.

## Implicit and explicit signals

For the current traffic situation, combining implicit signals based on driving behavior with explicit signals based on eHMI is a promising approach^28^. The signals provided by the vehicle to the pedestrian are divided into explicit and implicit signals. The eHMI is the explicit signal, while the implicit signal is the kinematic characteristics of the vehicle, such as speed, acceleration, separation distance and arrival time^29^. In a virtual study, participants interacted with a self-driving car with an externally mounted 360° LED light bar that displayed different signals depending on the kinematic characteristics of the vehicle. The results showed that the overall communication effect had great potential when the signal expression of the eHMI was aligned with the vehicle’s kinematic state. However, the findings also highlighted that when the signaling of the eHMI contradicted the behavior of the vehicle, the consequences could be fatal^30^. Moreover, when a vehicle has an eHMI, humans may ignore the actual movement state of the vehicle and focus instead on the signals presented by the eHMI^29^. Therefore, the key issue in effectively presenting vehicle information to pedestrians is how to efficiently combine explicit and implicit signals.

Most previous studies observed that front signal light only convey implicit signals when vehicles slow down^30^. In reality, however, many drivers do not slow down in front of zebra crossings under unsignalized roadways or even pass at a constant speed or accelerate to indicate that they are not yielding. In some traffic situations, vehicles conveying the intention not to yield to pedestrians can significantly speed up traffic flow^31^. In other words, it is not sufficient for a moving vehicle to communicate a deceleration signal to pedestrians; it is also necessary to communicate information when the vehicle is in other states of motion, such as constant speed or acceleration. This is also applicable to a wider range of road scenarios, such as pedestrians preparing to cross the road at an unsignaled zebra crossing while a vehicle is coming, vehicles going straight through the intersection horizontally and vertically at an unsignaled intersection, and two vehicles going in the same direction, one of which is preparing to overtake the other. Based on the above, this study attempts to communicate vehicle acceleration information using front signal light to convey vehicle movement intentions to pedestrians in an easily understood and natural way.

## Method

Two experiments were conducted in this study to investigate how the combination of a vehicle’s invisible signal (vehicle acceleration) and an explicit signal (front signal light) affects its interaction with pedestrians and to assess pedestrians’ affective response and satisfaction with this communication device.

Experiment I was conducted as a controlled test track in a real scenario to assess the effect of the use of acceleration signal light and different states of vehicle movement on the perceived behavior and emotional experience of pedestrians. Experiment II used a video-based controlled test track experiment and added two variables to Experiment I to extend the experiment to more complex road scenarios. The added variables were vehicle speed and day and night light conditions. The aim of Experiment II was to better consider the impact of complex road variables on the pedestrian-vehicle interaction experience. Experiment II could compensate for the lack of realism and acoustic cues present in Experiment I and validate the findings.

### Participants

A total of 77 participants were enrolled in two experiments of this study. Experiment 1 had 33 participants, including 17 males and 16 females, aged between 18 and 45 years (M = 24.03, SD = 4.384), and Experiment 2 had 44 participants, including 16 males and 28 females, aged between 18 and 26 years (M = 20.73, SD = 2.296). The subjects all had normal or corrected visual acuity. The participants’ mode of travel was mainly walking (64%) or public transport (49%). All participants provided written informed consent and were given experimental gifts.

### Design of acceleration signal light

**Figure 2:**
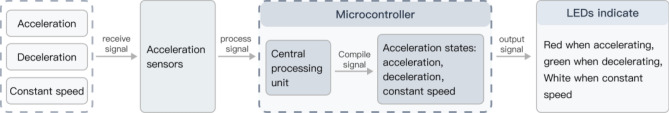
Schematic design of acceleration signal light.

**Figure 3:**
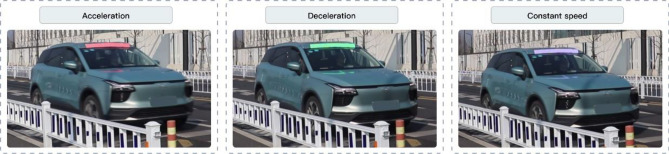
Schematic design of acceleration signal light.

In this study, an acceleration signal was designed to be applicable to both manually driven vehicles and autonomous vehicles. The hardware design of the acceleration signal light is shown in Fig. 2. The design principle is that the vehicle’s motion is captured by an acceleration sensor and sent to the central processor, which is then displayed on the signal light on top of the vehicle. The acceleration signal light are red, green and white when the vehicle is accelerating, decelerating and at constant speed, respectively (as shown in Fig. 3). The color of the acceleration signal light display is referenced from a study by Tibor Petzold et al.^7^, where red light convey a warning signal from a pedestrian perspective, green light represent a passing signal, and white light signal that the vehicle is in motion. This study only focused on whether the signals were clearly visible and did not consider industry guidelines or official regulations regarding signal colors.

### Experimental scenarios

**Figure 4:**
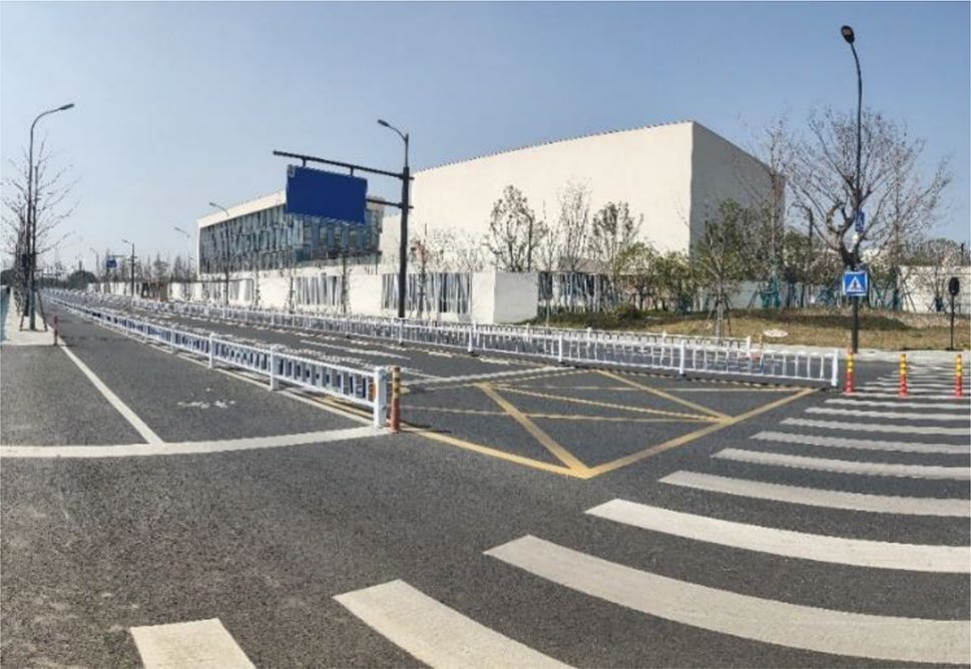
Schematic of the experimental section.

A partially closed roadway without signals in both directions and with pedestrian crossings was chosen as the experimental scenario for this study (as shown in Fig. 4). All participants completed the test on the same day. The experimental times were 10:00 am to 5:00 pm and 7:00 pm to 10:00 pm.

**Figure 5:**
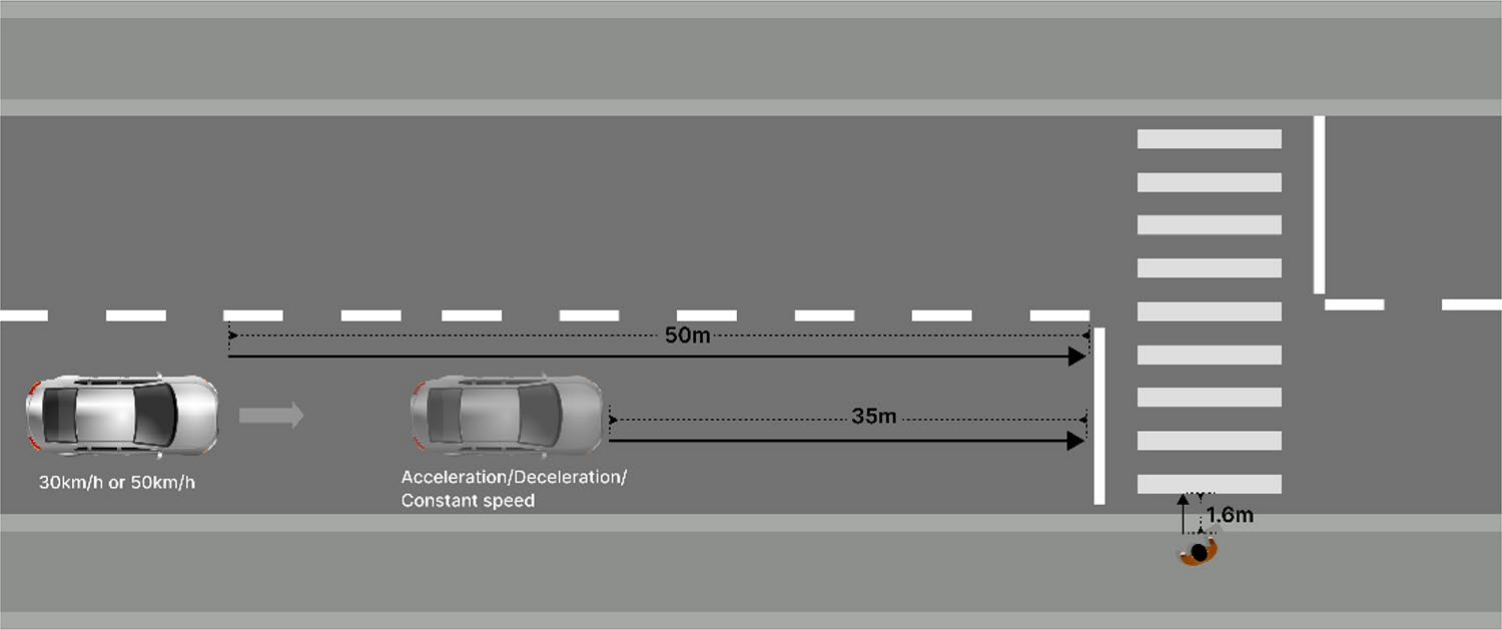
Experimental schematic.

As shown in Fig. 5, participants in Experiment 1 were asked to stand 1.60 m outside the crosswalk and observe the motion of the experimental vehicle. An experimenter driving a medium-sized vehicle (electric drive) entered the experimental roadway while traveling up to 50 m from the crosswalk and began to maintain the experimental vehicle speed, traveling up to 35 m from the crosswalk and began to change the vehicle’s state of motion (the purpose of this was to record the time at which the participant judged the change in the vehicle’s state of motion). There were three changes in the state of motion of the vehicle: (1) deceleration, slow deceleration to a stop at 5 m from the road; (2) constant speed through the sidewalk; (3) acceleration, slow acceleration across the sidewalk.

Experiment I was conducted as a field experiment, and the following provisions were adopted for the safety of pedestrians: (1) drivers were instructed to maintain a safe distance from pedestrians at all times; (2) no other vehicles or pedestrians were present in the test site during the experiment; (3) participants only stood at the zebra crossing and were not required to cross the road.

The test video for Experiment II was recorded with a camera (Canon 200d) with a full HD resolution of 1920 × 1080 pixels at 60 frames per second. To simulate the perspective of a pedestrian preparing to cross the road, the camera was placed 1.60 m outside the zebra crossing and 1.65 m above the ground. The final test video was edited using video editing software (Adobe Premiere), and the start of each video was set to 50 m before the vehicle passed the zebra crossing to record the time at which the participant became aware of the change in vehicle status. The participants used a 27′′ computer monitor to watch the test videos.

### Experimental design

Experiment I was a controlled test track experiment based on a real road scenario with 2 variables. Variable one was whether the vehicle used acceleration signal light: vehicles that used acceleration signal light were set as the experimental group, and vehicles that did not use acceleration signal light were set as the control group. Variable two was the vehicle motion state, which presented three common vehicle motions, i.e., deceleration, constant speed and acceleration. The experiment was conducted to reflect real traffic conditions, and the vehicle speed was not controlled, with the effect of slow acceleration or slow deceleration presented. Each tester participating in Experiment I was required to observe the approach of the experimental vehicle six times and judge the change in vehicle state.

Experiment II was based on viewing videos of vehicles approaching pedestrians in a controlled test track with the aim of enriching the interaction between pedestrians and vehicles on a more complex and comprehensive road. According to a study by Petzoldt et al.^7^, vehicle speed also has an effect on the degree of recognition of vehicle state. Therefore, the experimental vehicle driving speed was confirmed as slow (30 km/h) and fast (50 km/h), and it met the experimental road requirements. This study was designed to complement existing studies that have not explored the psychological aspects of different lighting conditions on pedestrians’ judgment of vehicle status^32,33^ and to further investigate the effect of day and night light conditions on pedestrians’ precrossing emotional experience. Therefore, a total of four variables were presented in Experiment II, vehicle speed, day and night light conditions, change in vehicle movement state and acceleration signal light use, for a total of 24 different vehicle states. The experimental group was vehicles with acceleration signal light and the control group was vehicles without acceleration signal light, each with 12 videos. Therefore, the participants in Experiment II were required to watch a total of 24 videos of different variables.

In both Experiment I and Experiment II, each participant was randomly selected to be tested in either the experimental or control group prior to the start of the experiment. The participant’s task was to press the stopwatch for the first time when the vehicle in the field or video entered the experimental section, followed by pressing the stopwatch a second time as soon as a change in the vehicle’s movement state was recognized, recording the time at which the change in the vehicle’s state was recognized and the result of the judgment. After each on-site observation of a vehicle or viewing of a video of a vehicle, participants were asked to complete the appropriate questionnaire (as shown in Table 1). The questionnaire was based on an assessment of the pedestrian’s willingness to cross the street and his or her emotional experience. At the end of the questionnaire, participants were also asked to rate their overall satisfaction with the acceleration signal light^34^ (as shown in Table 2). The scoring mechanism was a 5-point Likert scale (1 = “disagree” to 5 = “agree”).

**Table 1:** Participants' assessment of their willingness to cross the road, their trust and their sense of safety.

Assessment	Questionnaire
Willingness	Would you be willing to cross the road with this car in motion?
Perceived safety	Do you think the behavior of the vehicle enhances your sense of security?
Trustworthiness	Are you confident that you can effectively predict a vehicle's intentions?

**Table 2:** Participants’ assessment of overall satisfaction with acceleration signal light.

Assessment	Questionnaire
Question 1	Can you understand the connection between the different light colors and the state of the vehicle?
Question 2	Are you satisfied with the signal indicating the acceleration status of your vehicle?
Question 3	Do you think vehicle acceleration signals are more helpful for pedestrians to interact with vehicles?
Question 4	Would you like to use it in your own car or recommend it to others?
Question 5	Would you prefer to interact with vehicles on the roads of the future with directional signals?

### Ethical approval

The study was conducted in accordance with the Declaration of Helsinki, and approved by the Ethics Committee of School of Art and Design, Zhejiang Sci-Tech University.

## Results

### Experiment I

Experiment 1 used a two independent samples t-test to investigate the effect of the presence or absence of acceleration signal light on pedestrians’ perceived behavior and emotional experience under different states of vehicle motion (acceleration, deceleration, and constant speed). In this study, the data were tested for normal distribution assumptions (S-W test and histogram). The test results found that the data for recognition time conformed to normal distribution, so the two independent samples t-test was used, in addition, Levene’s variance alignment was tested, and the t-test results were used when the variance was assumed to be aligned, and the corrected t-test results were used when the variance was assumed to be not aligned. Whereas the data on pedestrian crossing willingness, safety perceptions and trust were not normally distributed, the non-parametric test (Mann–Whitney U test) was used.

#### Perceived behavioral assessment of pedestrians

Experiment I began with an analysis of pedestrian recognition of different states of motion of vehicles in the experimental and control groups. In the control group, participants correctly identified vehicle acceleration at 67%, vehicle deceleration at 94% and vehicle constant speed at 76%. In contrast, in the experimental group, the addition of the acceleration signal light significantly improved the correct pedestrian recognition rate, with 100% correct recognition of vehicle acceleration, 97% correct recognition of vehicle deceleration and 97% correct recognition of vehicle at a constant speed. Three participants made their judgments before the vehicle state actually started to change, and these data were removed from further analysis.

As shown in Table 3, there was a significant effect of whether the vehicle used acceleration signal light on the recognition time of pedestrians when the vehicles were accelerating (*p* < 0.001) and decelerating (*p* = 0.002), and the pedestrians recognized the experimental group vehicles significantly faster than the control group vehicles. In contrast, the difference in recognition time between the experimental and control vehicles was not significant when the vehicles were at constant speed (*p* = 0.222), probably because the acceleration signal light were white and pedestrians did not perceive this significantly.

**Table 3:** Two independent samples t-test for pedestrian recognition time.

	Groups	M (SD)	t	*p*	95% CI of difference
Acceleration	without acceleration signal light	3.52 (1.88)	4.521	< 0.001	1.12~2.99
	With acceleration signal light	1.47 (0.78)			
Deceleration	without acceleration signal light	3.35 (1.53)	3.259	0.002	0.50~2.14
	With acceleration signal light	2.03 (0.97)			
Constant speed	without acceleration signal light	3.81 (1.51)	1.242	0.222	− 0.42~1.75
	With acceleration signal light	3.15 (1.85)			

According to Table 4, there was a significant effect of whether the vehicle used acceleration signal light on pedestrians’ willingness to cross the street when the vehicles were accelerating (*p* = 0.006) and decelerating (*p* = 0.043). When the vehicle was accelerating, participants’ willingness to cross the street was lower than that of the control group vehicles in the experimental group, while when the vehicle was decelerating, participants’ willingness to cross the street was higher than that of the control group vehicles in the experimental group. Overall, the inclusion of acceleration signal light can help pedestrians to efficiently and accurately determine the state of vehicle movement, enhancing pedestrian safety on the road.

**Table 4:** Mann–Whitney U test for pedestrian crossing willingness.

Groups		M (P_25_,P_75_)	Mann–whitney U test	
			Z	*p*
Acceleration	without acceleration signal light	2.00 (2.00, 3.00)	− 2.894	0.006
	With acceleration signal light	1.00 (1.00, 2.00)		
Deceleration	without acceleration signal light	4.00 (3.25, 4.00)	− 2.452	0.043
	With acceleration signal light	4.00 (4.00, 5.00)		
Constant speed	without acceleration signal light	2.00 (2.00, 3.75)	− 0.680	0.529
	With acceleration signal light	3.00 (2.00, 4.00)		

#### Assessment of the emotional experience of pedestrians

The statistical analysis in Table 5 shows that there was a significant effect of whether the vehicle used acceleration signal light on pedestrians’ perception of safety in the state of vehicle deceleration (*p* = 0.003) constant speed (*p* = 0.016), and subjects’ perception of safety in the experimental group vehicles approaching is higher than that in the control group vehicles. Whereas, the use of acceleration signal light had a smaller increase in the safety perception of pedestrians when the vehicle was in the accelerated state.There is a significant difference between whether or not to use the acceleration signal light on the trust of pedestrians in the state of vehicle acceleration (*p* < 0.001) and deceleration (*p* < 0.001), and the subjects have a higher level of trust of the vehicle in the experimental group than the vehicle in the control group when the vehicle is approaching. In contrast, the use of acceleration signal light in te constant speed state of the vehicle resulted in a smaller increase in trust of pedestrians. Overall, the use of acceleration signal light in vehicles can improve pedestrians’ perception of safety and trust in vehicle behavior and enhance the perception of the pedestrian-vehicle interaction experience at a psychological level.

**Table 5:** Mann–Whitney U test for pedestrian safety perceptions and trustworthiness.

Groups		Security perception			Trust degree		
		M(P_25_,P_75_)	Mann–Whitney U test		M(P_25_,P_75_)	Mann–Whitney U test	
			Z	*p*		Z	*p*
Acceleration	without acceleration signal light	2.00 (2.00, 2.75)	− 0.979	0.328	2.00 (2.00, 3.75)	− 3.635	< 0.001
	With acceleration signal light	2.50 (1.25, 4.00)			4.00 (3.25, 5.00)		
Deceleration	without acceleration signal light	3.50 (3.00, 4.00)	− 2.933	0.003	4.00 (3.00, 4.00)	− 3.811	< 0.001
	With acceleration signal light	4.00 (4.00, 5.00)			4.00 (4.00, 5.00)		
Constant speed	without acceleration signal light	3.00 (2.00, 300)	− 2.418	0.016	3.00 (2.00, 3.75)	− 1.760	0.096
	With acceleration signal light	4.00(2.00, 4.00)			3.50(3.00, 4.00)		

#### Satisfaction assessment of acceleration signal light

As shown in Table 6, the participants’ mean scores were greater than 4 for all four assessment perspectives of the acceleration signal, but the participants rated their experience of using the acceleration signal slightly lower (M = 3.64, SD = 0.96), possibly due to traffic regulations and personal habits. The participants gave the highest ratings to their understanding of the correlation between acceleration signal color change and vehicle status, suggesting that the light color set in this study is reasonable for showing changes in vehicle status.

**Table 6:** Rating of pedestrian satisfaction with accelerated signal light.

Questionnaire	M	SD
1. Can you understand the connection between the different light colors and the state of the vehicle?	4.30	0.68
2. Are you satisfied with the signal indicating the acceleration status of your vehicle?	4.12	0.96
3. Do you think vehicle acceleration signals are more helpful for pedestrians to interact with vehicles?	4.06	0.90
4. Would you like to use it in your own car or recommend it to others?	3.64	0.96
5. Would you prefer to interact with vehicles on the roads of the future with directional signals?	4.21	0.89

### Experiment II

Experiment II used a 2 × 3 × 2 × 2 repeated-measures ANOVA to investigate the effects of four variables on the perceived behavior and emotional experience of pedestrians. The presence or absence of acceleration signal light was the between-group variable, and the vehicle movement state (acceleration, deceleration, constant speed), approach speed (30 km/h, 50 km/h) and lighting conditions (daytime, nighttime) were the within-group variables. The data were tested for a hypothesis of normal distribution in this study and conformed to a normal distribution. Furthermore, the data were checked for sphericity, which was either given or adjusted with Huynh–Feldt correction^35^. For significant main effects and interactions, pairwise comparisons with Bonferroni correction were calculated and reported. The effect size was reported with a partial eta quadrat (ƞp2). According to Cohen, the limits for the effect size were 0.01 (small effect), 0.06 (medium effect), and 0.14 (large effect)^36^.

#### Perceived behavioral assessment of pedestrians

Experiment II also began by analyzing the recognition of different states of motion of vehicles in the pedestrian control and experimental groups: in the control group, participants correctly identified 59% of vehicles accelerating, 77% of vehicles decelerating and 68% of vehicles at constant speed. While in the experimental group using acceleration signal light, 86% of vehicles in the accelerating state were correctly identified, 90% of vehicles in the decelerating state were correctly identified, and 86% of vehicles in the constant speed state were correctly identified. Experiments I and II were highly consistent in terms of the acceleration signal light improving the correct recognition of vehicle status by pedestrians. However, compared to Experiment I, pedestrians identified the vehicle status of the control group with a higher error rate. The reason for this may be that the video experiment was missing some realism and acoustic cues from the vehicles. One participant made a judgment before the vehicle state actually started to change, and the relevant data recorded for this participant were removed in further analysis.

As shown in Table 7, the use or nonuse of acceleration signal light by vehicles had a significant effect on the time it took for pedestrians to recognize a change in vehicle movement status (*p* < 0.001), which is consistent with the results of Experiment I. Based on Experiment I, further conclusions were drawn from Experiment II. First, there was a significant main effect (*p* < 0.05) of vehicle speed and day and night light conditions on pedestrians’ recognition time in judging changes in vehicle motion state; the slower the vehicle was traveling when approaching pedestrians or the darker the environment, the less likely pedestrians were to recognize the vehicle motion state. Second, there was a significant interaction effect between the presence or absence of acceleration signal light, vehicle travel speed when approaching pedestrians and day and night light conditions (F = 15.591, *p* < 0.001, ηp2 = 0.314). Simple effects analysis showed that participants identified the experimental group vehicles significantly faster than the control group vehicles when the vehicles were traveling at the same speed, either during the day or at night (*p* < 0.001). There was also a significant interaction effect between the use of acceleration signal light, the vehicle movement state and day and night light conditions (F = 4.788, *p* = 0.014, ηp2 = 0.123). Simple effects analysis showed that participants identified vehicles in the experimental group using acceleration signal light significantly faster than vehicles in the control group under the same light conditions regardless of whether the vehicles were accelerating or decelerating (*p* < 0.001), while pedestrians showed less improvement in the judgment of vehicles in the experimental group at a constant speed.

**Table 7:** ANOVA of four variables on pedestrian recognition time and willingness to cross the street.

	Recognition time			Willingness		
	F	*P*	ƞp2	F	*P*	ƞp2
With or without acceleration signal light	24.510	< 0.001	0.419	1.650	0.208	0.046
Movement status	23.822	< 0.001	0.412	158.297	< 0.001	0.823
Speed	166.732	< 0.001	0.831	72.771	< 0.001	0.682
Day and night	5.875	0.021	0.147	0.407	0.528	0.012
With or without acceleration signal light* Speed *Day and night	15.591	< 0.001	0.314	0.038	0.846	0.001
With or without acceleration signal light *Movement status * Speed	2.145	0.126	0.059	5.790	0.006	0.146
With or without acceleration signal light* Movement status* Day and night	4.788	0.014	0.123	2.444	0.094	0.067
Movement status* Speed* Day and night	20.092	< 0.001	0.371	0.042	0.959	0.001
With or without acceleration signal light* Movement status* Day and night* Speed	0.175	0.840	0.005	4.195	0.021	0.110

According to Table 7, there was no significant effect of whether a vehicle used acceleration signal light on pedestrians’ willingness to cross the street, while there was a significant main effect of vehicle approach speed on pedestrians’ willingness to cross the street (*p* < 0.001). Specifically, as vehicles approached faster, pedestrians’ willingness to cross was lower. There was a significant interaction effect (F = 4.195, *p* = 0.021, ηp2 = 0.110) between the four factors of whether acceleration signal light were used, movement status, approach speed and day and night light conditions. Simple effects analysis revealed that participants were significantly less willing to cross the street for vehicles in the experimental group than for those in the control group when the vehicles accelerated at lower approach speeds during daylight conditions (F = 20.029, *p* < 0.001, ηp2 = 0.371). In contrast, there was no significant difference in the participants’ intention to cross the street in darkness.

#### Assessment of the emotional experience of pedestrians

As shown in Table 8, there was a significant effect of whether the vehicle used acceleration signal light on the perception of pedestrian safety (*p* < 0.001), with participants perceiving the experimental group as significantly safer than the control group of vehicles. There was a significant main effect of vehicle approach speed on pedestrians’ perception of safety (*p* < 0.001), with participants’ perception of safety decreasing with faster vehicle approach speed. There was a significant interaction effect (F = 4.235, *p* = 0.019, ηp2 = 0.111) between the factors of whether acceleration signal light were used, approach speed and movement state. Simple effects analysis revealed that participants perceived safety as significantly higher in the experimental group than in the control group when the vehicle was accelerating at a lower approach speed or accelerating at a higher approach speed and at a constant speed (*p* < 0.001).

**Table 8:** ANOVA of four variables on pedestrian affective experience.

	Security perception			Trust degree		
	F	*P*	ƞp2	F	*P*	ƞp2
With or without acceleration signal light	19.739	< 0.001	0.367	1.600	0.215	0.045
Movement status	82.899	< 0.001	0.709	31.707	< 0.001	0.483
Speed	40.780	< 0.001	0.545	0.536	0.469	0.016
Day and night	0.000	1.000	0.000	10.979	0.002	0.244
With or without acceleration signal light* Speed *Day and night	0.033	0.856	0.001	0.000	1.000	0.000
With or without acceleration signal light *Movement status * Speed	4.235	0.019	0.111	0.283	0.747	0.008
With or without acceleration signal light* Movement status* Day and night	2.265	0.114	0.062	0.218	0.790	0.006
Movement status* Speed* Day and night	1.901	0.167	0.053	0.292	0.733	0.009
With or without acceleration signal light* Movement status* Day and night* Speed	2.626	0.093	0.072	1.162	0.317	0.033

In terms of pedestrian trust in vehicle movement, the variable of whether the vehicle was using acceleration signal light did not cause a significant effect (as shown in Table 8). There was a significant interaction effect between whether acceleration signal light were used and the state of motion (F = 7.674, *p* = 0.001, ηp2 = 0.184). Simple effects analysis showed that pedestrians trusted the experimental group vehicles more than the control group vehicles in the vehicle acceleration state (F = 7.132, *p* = 0.012, ηp2 = 0.173).

#### Satisfaction assessment of acceleration signal light

As shown in Table 9, the participants’ mean scores for all five questions in the acceleration signal light satisfaction assessment were greater than 4. The participants’ scores for the feasibility of interaction with acceleration signal light were the highest, indicating that acceleration signal light are beneficial for pedestrians to communicate with vehicles, which is generally consistent with the results of Experiment I.

**Table 9:** Rating of pedestrian satisfaction with accelerated signal light.

Questionnaire	M	SD
1. Can you understand the connection between the different light colors and the state of the vehicle?	4.27	0.94
2. Are you satisfied with the signal indicating the acceleration status of your vehicle?	4.00	0.69
3. Do you think vehicle acceleration signals are more helpful for pedestrians to interact with vehicles?	4.27	0.77
4. Would you like to use it in your own car or recommend it to others?	4.09	0.87
5. Would you prefer to interact with vehicles on the roads of the future with directional signals?	4.36	0.49

Combining the results of Experiment I and Experiment II, it was verified that the use of acceleration signal light in vehicles helps to improve the experience of pedestrians when interacting with vehicles. The specific experimental results are as follows.

1. Both experiments demonstrated that when vehicles use acceleration signal light, pedestrians are more likely to judge the vehicle’s state of motion, improving pedestrians’ affective experience and enhancing pedestrians’ perception of safety and trust in the behavioral intentions of the vehicle, which in turn helps pedestrians to make safe street-crossing decisions.
2. Pedestrians recognize vehicle acceleration most easily, followed by deceleration; the most difficult to recognize is constant speed. Although the use of acceleration signal light marginally improves pedestrian recognition of the constant speed state of vehicles, it still falls short of the desired effect of efficient recognition of vehicle intentions.
3. The need to use acceleration signal light at different vehicle approach speeds and under different lighting conditions was demonstrated in Experiment II. Pedestrians’ willingness to cross and their perception of safety increased when vehicles approached at slower speeds, but they were less likely to recognize vehicle intentions when pedestrian-vehicle conflicts were likely to occur. The use of acceleration signal light by vehicles improved this problem. In nighttime conditions, acceleration signal light were more likely to improve pedestrians’ perception and trust in vehicles.
4. Participants rated the acceleration signal light as highly satisfactory, particularly in terms of their functionality and interactivity.

## Discussion

Both experiments conducted in this study demonstrate that acceleration signal light can play a positive role in pedestrian-vehicle interaction and that the results of Experiment I and Experiment II can be verified against each other. First, at the level of pedestrian perception behavior, both experiments show that the use of acceleration signal light by vehicles significantly improves the correct recognition rate and efficiency of pedestrians in recognizing vehicle acceleration and deceleration states and influences pedestrians’ willingness to cross the street. Recognition of a vehicle’s constant speed state is inherently difficult for pedestrians, and the acceleration signal light is not as effective as expected in indicating that the vehicle is at a constant speed. In other words, the focus for improving the efficiency of acceleration signal light recognition and the accuracy of information transfer could be the way in which the constant speed state of the vehicle is expressed. Overall, acceleration signal light communicate intuitive vehicle behavior intentions to pedestrians, making them aware of vehicle acceleration and deceleration and facilitating the generation of correct road decisions for pedestrians, thus optimizing the way pedestrians interact with vehicles and reducing the number of accidents. At the same time, pedestrians’ awareness of vehicle intentions facilitates reduced waiting times and a corresponding increase in traffic flow efficiency, resulting in safer and more efficient urban traffic^37^.

Second, at the level of pedestrians’ psychological perception, the use of acceleration signal light by vehicles also enhances pedestrians’ perception of road safety and trust in the behavior of vehicles, enhancing the emotional experience. Vehicles equipped with acceleration signal light are effective in enhancing pedestrians’ perception of safety on the road and deepening their trust in the vehicle’s intentions regardless of the vehicle’s changing state of motion. In particular, the use of acceleration signal light increases pedestrian safety and trust when vehicles are approaching at high speeds or when there is insufficient light. This positive emotional experience further promotes proper decision-making by pedestrians in road traffic, improving the efficiency and safety of the overall traffic system^38^. Acceleration signal light can thus create a better interaction mechanism between vehicles and pedestrians and promote harmonious coexistence in road traffic.

Finally, this study addresses satisfaction with acceleration signal light. Satisfaction with acceleration signal light was divided into three areas, functionality, interactivity and usability. Pedestrians were more satisfied with the functionality and interactivity of acceleration signal light. Specifically, the information conveyed by the acceleration signal light was easy to understand and facilitated interactive communication between vehicles and pedestrians. In terms of usability, while pedestrians were generally positive about the experience of using acceleration signal light, they still had doubts about their future application. In summary, acceleration signal light have significant advantages in clearly communicating vehicle information, enhancing pedestrian-vehicle interactions and improving road safety and can continue to be optimized to increase the likelihood of realistic applications and provide a new solution for improving urban road safety^39^.

The interaction between vehicles’ explicit signals (front signal light) and vehicles’ invisible signals (vehicle acceleration states) can effectively communicate vehicles’ behavior and intentions, positively guide pedestrians in their crossing decisions and initially achieve the research objective of enhancing traffic road safety. Studying the way in which explicit and implicit vehicle signals work together to convey vehicle information has proven to be an innovative direction in the field of traffic safety that can facilitate rapid road decisions by pedestrians^33^. In contrast, research on the effects of vehicle speed and lighting conditions on vehicle information transmission is a common research direction in the field of traffic. In this study, the lower the speed at which the vehicle was traveling, the more positive and efficient the pedestrian’s behavior was in terms of road decision-making and the higher the sense of psychological safety. This corresponds to the finding that pedestrians have shorter reaction times when faced with low-speed and high-deceleration-rate vehicles^7^. Day and night conditions have less influence on pedestrians’ judgment of vehicle status, which is similar to the findings of Rothenbücher et al.^40^. Overall, the design and application of acceleration signal light may become a new research idea in the field of pedestrian-vehicle interaction.

## Conclusion

This study finds that acceleration signal light can improve the current situation of human-vehicle conflict and provide an effective solution for creating a safe road environment. The solution combines the implicit signals of vehicles with explicit signals to transmit information to pedestrians about the change in the vehicle movement status by means of light color change, improving the communication experience between the vehicle and pedestrians and achieving the purpose of maintaining traffic safety. When vehicles are moving faster, acceleration signal light can improve pedestrians’ judgment of a vehicle’s movement status, making it easier to make the right decision and significantly increasing pedestrians’ perception of safety and trust in the vehicle. In addition, the light display has a positive effect on the emotional experience of pedestrians in the nighttime environment and contributes to the maintenance of traffic at night. Acceleration signal light, as a type of front signal light device that can efficiently communicate vehicle information, will play an important role in the future in creating a safer, more efficient and more comfortable road environment for people.

This study demonstrates that acceleration signal light are a viable proposal that can improve the pedestrian-vehicle interaction experience; however, this conclusion requires further validation. First, in terms of the design of acceleration signal light, the color, brightness, shape or position of the signal in front of the vehicle needs further investigation to improve the prominence and comprehensibility of these light. Furthermore, the way in which acceleration signal light convey information about the vehicle’s constant speed status needs to be further explored to give pedestrians a fuller picture of vehicle behavior. Second, in terms of experimental design, the participants in this study were all students on campus, while the road pedestrians included all age groups. Therefore, the population sample lacked richness. The experimental road scene environment was also relatively uniform. Pedestrians’ recognition of acceleration signal light may be affected if the environment is obscured by other vehicles or involves a large number of light sources that increase the complexity of the environment.

In the experiment, there may also be a situation where the vehicle was slowing down and the acceleration signal gave a signal, but the vehicle was unable to stop within a safe distance, making it impossible for pedestrians to cross the street safely. There are three possible reasons for this: insufficient braking distance due to excessive speed, poor road conditions, and vehicle failure. In future research, the design and performance of the acceleration signal light can be further explored to optimize its design or integration with other smart hardware and software to enhance the safety of pedestrians on the road.

## Data Availability

The datasets used and/or analysed during the current study available from the corresponding author on reasonable request.
